# Coiled-coil-mediated dimerization of Atg16 is required for binding to the PROPPIN Atg21

**DOI:** 10.1098/rsob.230192

**Published:** 2023-11-22

**Authors:** Miranda Bueno-Arribas, Celia Cruz-Cuevas, María-Angeles Navas, Ricardo Escalante, Olivier Vincent

**Affiliations:** ^1^ Instituto de Investigaciones Biomédicas Sols-Morreale CSIC-UAM, Madrid, 28029, Spain; ^2^ Departamento de Bioquímica y Biología Molecular, Facultad de Medicina, Universidad Complutense de Madrid, Madrid, Spain

**Keywords:** autophagy, proppin, WIPI, ATG21, ATG16, reverse two-hybrid

## Abstract

PROPPINs/WIPIs are β-propeller proteins that bind phosphoinositides and contribute to the recruitment of protein complexes involved in membrane remodelling processes such as autophagosome formation and endosomal trafficking. Yeast Atg21 and mammalian WIPI2 interact with Atg16/ATG16L1 to mediate recruitment of the lipidation machinery to the autophagosomal membrane. Here, we used the reverse double two-hybrid method (RD2H) to identify residues in Atg21 and Atg16 critical for protein–protein binding. Although our results are generally consistent with the crystal structure of the Atg21-Atg16 complex reported previously, they also reveal that dimerization of the Atg16 coiled-coil domain is required for Atg21 binding. Furthermore, most of the residues identified in Atg21 are conserved in WIPI2 and we showed that these residues also mediate ATG16L1 binding. Strikingly, these residues occupy the same position in the β-propeller structure as residues in PROPPINs/WIPIs Hsv2 and WIPI4 that mediate Atg2/ATG2A binding, supporting the idea that these proteins use different amino acids at the same position to interact with different autophagic proteins. Finally, our findings demonstrate the effectiveness of the RD2H system to identify critical residues for protein–protein interactions and the utility of this method to generate combinatory mutants with a complete loss of binding capacity.

## Graphical abstract

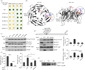

## 1. Introduction

Macroautophagy (hereafter autophagy) is a cellular degradation process conserved in eukaryotes and initially characterized in the model yeast *Saccharomyces cerevisiae*. Two types of autophagy have been described: selective and non-selective ‘bulk’ autophagy. Bulk autophagy mediates the nonspecific degradation of cytosolic components in response to nutrient deprivation and is necessary to maintain cellular homeostasis, while selective autophagy targets specific cargoes such as damaged organelles or protein aggregates. Both processes involve autophagy-related proteins (ATG), which are conserved from yeast to mammals. These proteins act in sequential steps to induce the formation of a double membrane structure, the phagophore, which engulfs the cargo to be degraded [1,2]. The resulting vesicle called autophagosome then fuses with the vacuole/lysosome, leading to degradation of its contents.

In bulk autophagy, nutrients starvation triggers the assembly of the Atg1/ULK initiation complex at the phagophore assembly site (PAS), located in close proximity to the endoplasmic reticulum (ER) [3]. Atg1/ULK-mediated activation of phosphatidyl-inositol-3-kinase complex 1 generates phosphatidyl-inositol-3-phosphate (PtdIns3P) in the phagophore membrane [4]. PtdIns3P-mediated recruitment of proteins called PROPPINs (beta-propellers that bind phosphoinositides) in yeast and WIPIs (WD40 repeat-containing proteins that interact with phosphoinositides) in mammals contributes to the recruitment of other ATG proteins involved in autophagosome elongation and lipidation [5,6]. Notably, mutations in the WIPI genes are associated with several neurological disorders [5,6].

PROPPINs/WIPIs fold as seven-bladed β-propellers. Three PROPPINs have been identified in yeast: Atg18, Hsv2 and Atg21 [7–11]. Atg18 plays an essential role in phagophore elongation through its association with Atg2, which mediates the transfer of phospholipids from the ER to the autophagosomal membrane [12–15]. Although Hsv2 also binds Atg2, it is only partially required for micronucleophagy, a type of selective autophagy [11,16]. The third PROPPIN, Atg21, interacts with Atg16 and enables the recruitment of the E3-like Atg12-Atg5-Atg16 complex of the lipidation machinery, which catalyzes the conjugation of Atg8 to phosphatidylethanolamine at the autophagosomal membrane [17–19]. Atg21 is essential for selective autophagy but is not absolutely required for bulk autophagy, as there are alternative mechanisms for the recruitment of the E3 complex [9,20–22]. In mammals, there are four WIPI proteins called WIPI1, WIPI2, WIPI3/WDR45B and WIPI4/WDR45. Like Atg18, WIPI3 and 4 interact with ATG2A/B and are involved in phagophore elongation [23–30], while the functional counterpart of Atg21, WIPI2, binds to ATG16L1 and is involved in the lipidation process, possibly in combination with WIPI1 [31–34].

Studies in yeast have shown that Atg21 binds to the coiled-coil domain (CCD) of Atg16 that mediates protein dimerization [17,35]. Atg16 also contains a membrane-binding amphipathic helix at the C-terminal end [36] and an Atg5-binding domain in the N-terminal region that mediates its interaction with the Atg12-Atg5 conjugate and the formation of the E3-like Atg12-Atg5-Atg16 complex [37]. In mammals, ATG16L1 also contains a CCD, an ATG5-binding domain and lipid-binding motifs [38–41]. However, it is a much larger protein than its yeast homologue as it harbours a C-terminal extension with a WD40 repeat domain involved in non-canonical autophagy [42]. In addition, it contains a RAB33 binding site in the CCD [43–45], and binding sites for WIPI2 and the scaffolding subunit of the initiation complex FIP200, in the region immediately downstream of the CCD, which is missing in the yeast protein [31,46,47].

The aim of this work was to identify the amino acid residues in Atg21 and Atg16 that play a critical role in protein–protein interaction by using the recently developed reverse double two-hybrid method (RD2H) [48]. Overall, our findings are in agreement with the recently reported crystal structure of Atg21 bound to Atg16 CCD [19], but unexpectedly our results also indicate that CCD-mediated dimerization of Atg16 is required for its interaction with Atg21. Furthermore, we found that the same residues in Atg21 and its human homologue WIPI2 mediate the interaction with Atg16 or ATG16L1. Our findings, together with previous studies, support the idea that proteins of the PROPPIN/WIPI family use different amino acid residues occupying the same position in the beta-propeller structure to interact with either Atg16/ATG16L1 or Atg2/ATG2A.

## 2. Results

### 2.1. Identification of Atg21 residues that mediate Atg16 binding

**Figure 1.**
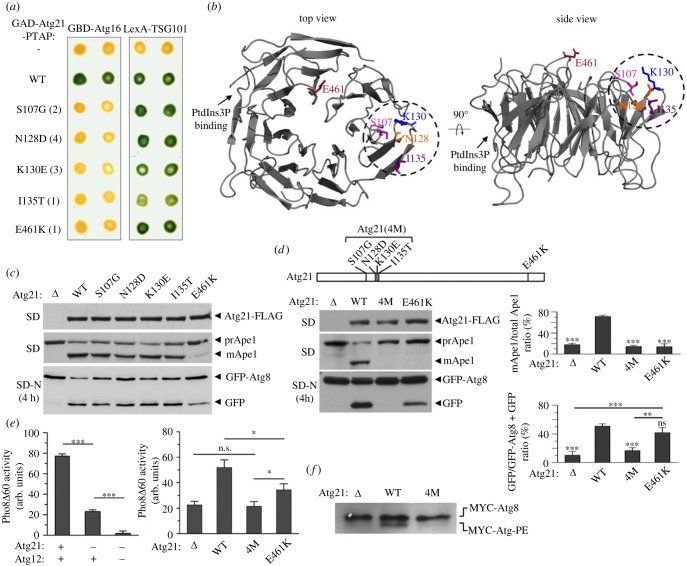
Identification and characterization of mutations in Atg21 that disrupt its interaction with Atg16. (*a*) Two-hybrid analysis of Atg21 mutants isolated in a reverse two-hybrid screen. GAD-Atg21-PTAP fusions containing the indicated amino acid substitutions were tested for two-hybrid interaction with GBD-Atg16 or LexA-TSG101 in strains Y187 or CTY10-5d, respectively. Interactions were revealed by β-galactosidase lift filter assays. Numbers in parentheses indicate the number of times the mutation was identified in the screen. (*b*) Localization of the mutated residues in the 3D structure of Atg21. Atg21 structure was predicted using Robetta (http://robetta.bakerlab.org) based on the structure of Atg21 from *Kluyveromyces lactis* (PDB: 6RGO). Top and side views of Atg21 structure were generated with PyMOL software (The PyMOL Molecular Graphics System, version 2.4.0 Schrödinger, LLC). Mutated residues are shown as coloured sticks and neighbouring residues are marked with a circle. An arrow indicates the PtdIns3P binding site. (*c*) Effect of Atg21 mutations on autophagy. OVY381 (*atg21Δ*) was cotransformed with pGFP-Atg8 and either pAtg21-FLAG (WT), the indicated mutant derivatives or an empty vector (*Δ*). Cells were grown to mid-log phase (SD) or starved 4 h in SD-N medium (SD-N). Protein extracts were immunoblotted with anti-Flag to detect Atg21-FLAG, anti-Ape1 to detect the precursor (prApe1) and mature form (mApe1) of Ape1, or anti-GFP to detect GFP-Atg8 and free GFP. (*d*) The same experiment as in C with a quadruple mutant (4 M) of Atg21. The positions of mutations in the quadruple mutant (4 M) are shown at the top. Two graphs representing the relative amount of mature Ape1 and free GFP for each mutant calculated from three independent experiments with standard deviation are shown on the right. Asterisks above error bars indicate significant differences with the strain carrying WT Atg21 as indicated by one-way ANOVA. **p > 0.01, ***p > 0.001. All significant differences are shown. A representative blot is shown on the left. (*e*) Pho8*Δ*60 assay. Left: OVY478 (*PHO8Δ60 atg21Δ atg12Δ*) was cotransformed with pAtg12-HA and pAtg21-FLAG, pAtg12-HA and pRS313, or pRS315 and pRS313. Right: OVY425 (*PHO8Δ60 atg21Δ*) was transformed with pAtg21-FLAG (WT), the indicated mutant derivatives or an empty vector (*Δ*). Cells grown to mid-log phase were starved 4 h in SD-N medium and the Pho8*Δ*60 assay was carried out as described in Material and Methods. The mean values are shown with standard deviation (*n* = 3). Only one difference was statistically non significant (ns) as indicated by one-way ANOVA. *p > 0.05, ***p > 0.001. (*f*) Atg8 lipidation analysis. OVY461 (*atg21Δ atg18Δ*) was cotransformed with pMYC-Atg8 and either pAtg21-FLAG (WT), the quadruple mutant derivative (4 M) or an empty vector (*Δ*). Cells were grown to mid-log phase in SD medium and starved 4 h in SD-N medium. Protein extracts were immunoblotted with anti-MYC to detect MYC-Atg8. Positions of MYC-Atg8 and lipidated MYC-Atg8-PE are indicated.

Previous studies have shown that Atg21 interacts with Atg16 and mediates the recruitment of the E3 complex Atg12-Atg5-Atg16 to the phagophore membrane [19]. In order to determine which residues in Atg21 are critical for binding Atg16, we performed a reverse double two-hybrid screen (RD2H) [48] to identify missense mutations in Atg21 that disrupt the interaction with Atg16. This method is based on generating random mutations in a fusion of Atg21 with the Gal4 transcriptional activation domain (GAD) and a PTAP motif-containing peptide at the N-terminal and C-terminal ends, respectively. A double reporter system allows the selection of mutants of the GAD-Atg21-PTAP fusion that have lost the ability to interact with a Gal4 DNA-binding domain (GBD) fusion to Atg16 but still interact with a LexA DNA-binding domain fusion to the PTAP-binding human protein TSG101. This selection eliminates all mutations that truncate the protein and thus eliminate the PTAP motif or destabilize the fusion. By using this method, we identified 5 missense mutations in Atg21, some repeatedly, that disrupt Atg16 binding in two-hybrid assays without preventing PTAP-mediated interaction with TSG101 (figure 1*a*). Localization of the mutated residues in the 3D structure of Atg21 shows that all five residues protrude from the top surface of the beta-propeller and that four of them are adjacent residues in blade 2 (Ser107, Asn128, Lys130 and Ile135) while one of them is further away in blade 7 (Glu461) (figure 1*b*).

We then expressed near-endogenous levels of the mutant proteins in a *Δatg21* strain to analyse the effect of these mutations on Atg21 function in autophagy. We analysed the processing of GFP-Atg8 to free GFP to monitor the progression of bulk autophagy after a switch to nitrogen-starvation medium (SD-N), and the processing of Ape1 in nutrient-rich medium (SD) to monitor selective autophagy [49,50]. Consistent with previous work [9], the precursor of Ape1 (prApe1) is not processed to the mature form (mApe1) in the *Δatg21* mutant, indicating a complete block of selective autophagy (figure 1*c*). Furthermore, processing of GFP-Atg8 to free GFP is strongly reduced compared with wild-type, demonstrating that bulk autophagy is also severely impaired (figure 1*c*). However, no defects in bulk or selective autophagy are detected in the mutants isolated in the reverse two-hybrid screen, with the exception of the E461K substitution, which strongly reduces selective autophagy and partially compromises bulk autophagy. In addition, and consistent with the selection method, none of these mutations appears to affect Atg21 stability (figure 1*c*). The involvement of several nearby residues of Atg21 blade 2 in binding to Atg16 raises the possibility that mutation of a single residue is not sufficient to prevent binding *in vivo*, which would explain the lack of autophagy phenotype of the mutants. The inhibitory effect of these mutations on two-hybrid interactions could be explained by the fact that this assay involves two fusion proteins in an isolated context, unlike *in vivo*, where these proteins are part of a complex involving additional contacts. To overcome this problem and ensure complete inactivation of the Atg16 binding site in blade 2, we generated a quadruple mutant of the nearby residues that we named Atg21(4M) (figure 1*d*). Analysis of autophagy shows that this quadruple mutant has an even greater effect than the E461K substitution, as we observed an inhibition of both selective and bulk autophagy similar to that of the *Δatg21* mutant (figure 1*d*). To quantify more precisely the effect of these mutations on bulk autophagy, we performed the alkaline phosphatase (ALP) enzyme assay, which is based on the use of a truncated alkaline phosphatase derivative (Pho8*Δ*60) that can only be transported in an autophagy-dependent manner to the vacuole for activation [50]. In agreement with previous work, bulk autophagy is not completely inhibited in the absence of Atg21 and fully blocked in the absence of both Atg21 and Atg12 (figure 1*e*, left graph). In addition, and consistent with the results obtained with GFP-Atg8, the E461K substitution in Atg21 partially reduces bulk autophagy, whereas the quadruple mutant has an identical effect as the *Δatg21* mutant, demonstrating that complete loss of Atg16 binding fully inactivates the function of Atg21 in autophagy (figure 1*e*, right graph). In addition, and in agreement with previous work [20], we showed that this inhibition of autophagy is associated with a decrease in lipidation of Atg8 (figure 1*f*).

### 2.2. Identification of Atg16 residues involved in Atg21 binding

**Figure 2.**
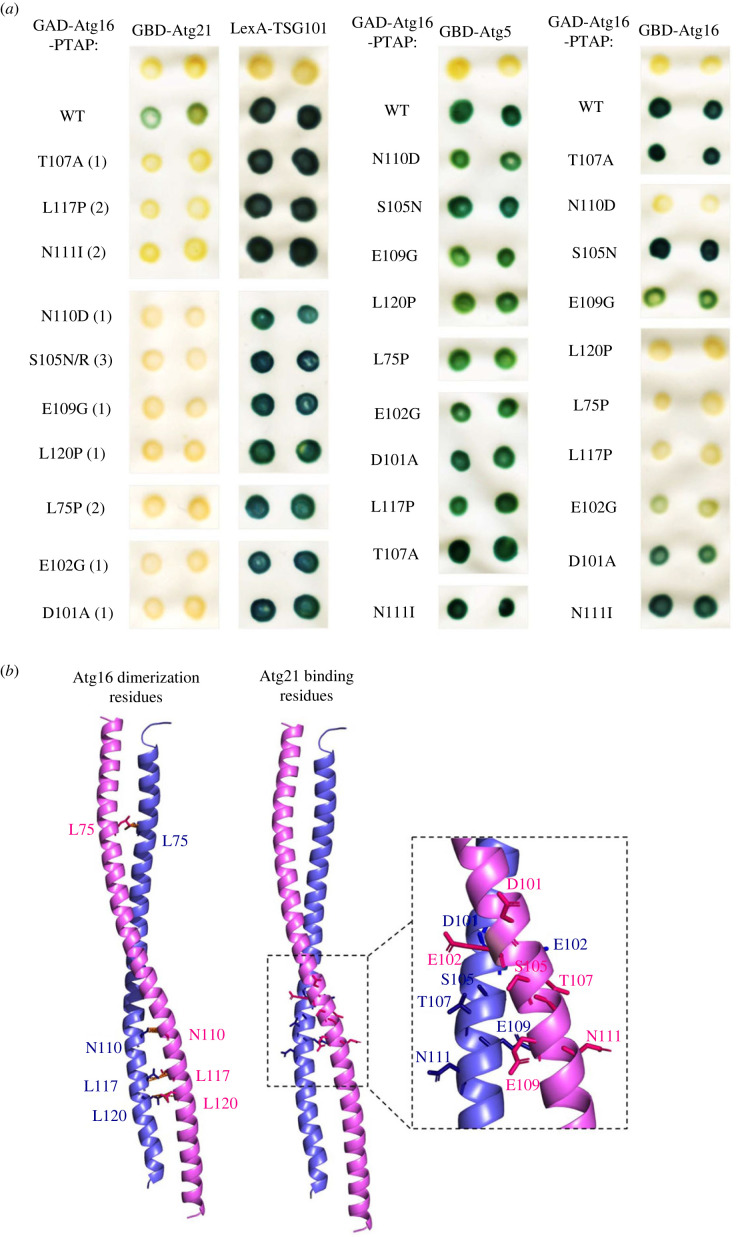
Identification of mutations in Atg16 that prevent binding to Atg21. (*a*) Two-hybrid study of Atg16 mutants isolated in a reverse two-hybrid screen. GAD-Atg16-PTAP fusions containing the indicated amino acid substitutions were assayed for two-hybrid interaction with GBD-Atg21, LexA-TSG101, GBD-Atg5 or GBD-Atg16 in strains Y187 (GBD) or CTY10-5d (LexA). Positive interactions were detected by β-galactosidase lift filter assays and mutants are ordered according to the order in which they were identified in the screen. Numbers in parentheses indicate the number of times the mutation was identified in the screen. (*b*) Localization of the mutated residues in the 3D structure of Atg16. Atg16 coiled-coil structure of *Saccharomyces cerevisiae* (PDB: 3A7P) was visualized by using PyMOL. Atg16 monomers are shown in pink and blue. Left: mutated residues that prevent Atg16 dimerization are shown as coloured sticks and bonding interactions as orange dotted lines. Right: mutated residues that only disrupt Atg16-Atg21 binding are shown as coloured sticks.

Reciprocally, we used the same approach to identify residues in Atg16 required for binding to Atg21. Here, we performed a RD2H screen to select randomly generated mutations in a GAD-Atg16-PTAP triple fusion that disrupt the interaction with GBD-Atg21 without preventing PTAP-mediated binding to LexA-TSG101. We identified ten missense mutations in Atg16, some also repeatedly, which disrupt the interaction with Atg21 without preventing binding to TSG101 (figure 2*a*, left columns). The effect of these mutations is specific, as they do not prevent the interaction of Atg16 with another protein of the E3 complex, Atg5 (figure 2*a*, middle column). Unexpectedly, we observed that four of these mutations also disrupt the interaction of Atg16 with itself, whereas others have no or only a partial effect (figure 2*a*, right column). Localization of the ten mutated residues in the three-dimensional structure of Atg16 shows that they all belong to the coiled-coil domain (CCD) of Atg16 (residues 58–123) [35]. Consistent with the two-hybrid results, the four mutated residues that disrupt the interaction of Atg16 with itself are located at the coiled-coil dimer interface (figure 2*b*, left) and are positioned at the a or d sites of the heptad repeats that enable dimerization [35]. The other six mutated residues are not involved in the dimeric coiled-coil structure and are nearby residues located in the middle of the CCD, between amino acids 101 and 111 (figure 2*b*, right). Two of them, Glu101 and Asp102, have been previously identified as being part of the Atg21 binding site [19].

**Figure 3.**
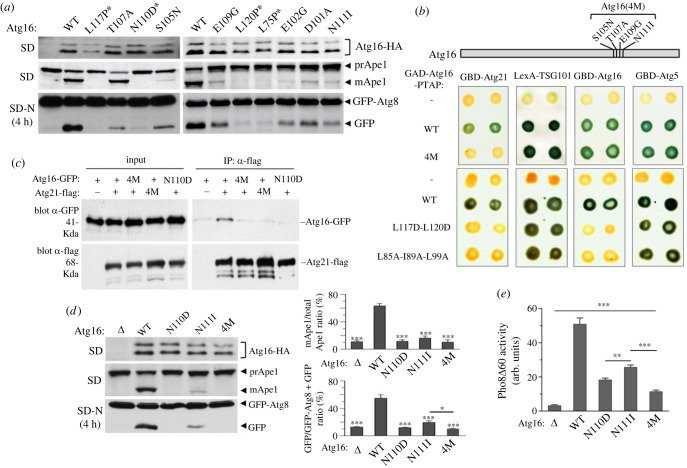
Functional analysis of mutations in Atg16 that disrupt Atg21 binding. (*a*) Effect of Atg16 mutations on autophagy. OVY383 (*atg16Δ*) was cotransformed with pGFP-Atg8 and either pAtg16-HA (WT), the indicated mutant derivatives or an empty vector (*Δ*). Cells were grown to mid-log phase (SD) or starved 4 h in SD-N medium (SD-N). Protein extracts were immunoblotted with anti-HA to detect Atg16-HA, anti-Ape1 to detect the precursor (prApe1) and mature form (mApe1) of Ape1, or anti-GFP to detect GFP-Atg8 and free GFP. Asterisks indicate mutations that prevent Atg16 dimerization. (*b*) Two-hybrid analysis of a quadruple mutant (4 M) and dimerization mutants of Atg16. The positions of mutations in the 4M mutant are shown at the top. The indicated GAD-Atg16-PTAP fusions were tested for two-hybrid interaction with GBD-Atg21, LexA-TSG101, GBD-Atg16 or GBD-Atg5 in strains Y187 (GBD) or CTY10-5d (LexA). Positive interactions were detected by β-galactosidase lift filter assays. (*c*) Coimmunoprecipitation of Atg16 and Atg21 from yeast cell extracts. OVY382 (*atg18Δ*) cotransformed with pmAtg21-FLAG (+) or vector control pRS425 (−) and pmAtg16-GFP (+) or the indicated mutant derivatives was grown to mid-log phase and treated with rapamycin for 120 min. Cross-linking reagent DSP was used prior to lysis and anti-Flag-immunoprecipitated protein extracts were immunoblotted with anti-GFP (90% of the total precipitates) or anti-Flag (10% of the total precipitates) Ab. Input represents 0.5% of protein extracts for Atg16-GFP and 5% for Atg21-FLAG. (*d*) Effect of the Atg16 quadruple mutation (4 M) on autophagy. Same experiment as in (*a*) with the indicated Atg16 mutant derivatives. Two graphs representing the relative amount of mature Ape1 and free GFP for each mutant calculated from three independent experiments with standard deviation are shown on the right. Asterisks above error bars indicate significant differences with the strain carrying WT Atg16 as indicated by one-way ANOVA. *p > 0.05, ***p > 0.001. All significant differences are shown. A representative blot is shown on the left. (*e*) Pho8*Δ*60 assay. OVY417 (*PHO8Δ60 atg16Δ*) was transformed with pAtg16-HA (WT), the indicated mutant derivatives or an empty vector (*Δ*). Cells grown to mid-log phase were starved 4 h in SD-N medium and the Pho8*Δ*60 assay was performed. The mean values are shown with standard deviation (*n* = 3). All differences are statistically significant (one-way ANOVA). **p > 0.01, ***p > 0.001.

To assess the effect of these mutations on autophagy, we expressed near-endogenous levels of the corresponding mutant proteins in a *Δatg16* strain and analysed Ape1 and GFP-Atg8 processing to monitor selective and bulk autophagy, respectively. We found that the four mutations at the dimer interface (L117P, N110D, L120P and L75P) block selective autophagy and strongly reduce bulk autophagy (figure 3*a*). L117P appears to have even a stronger inhibitory effect on bulk autophagy but the lower expression level of the mutant protein could contribute to this effect. The other six mutations that directly affect the Atg21 binding site have a lesser effect as they do not completely block selective autophagy and have a partial effect on bulk autophagy. One of these mutations, T107A, has the least effect, as it does not seem to affect selective autophagy. Following the same approach as above, and to ensure complete inactivation of the Atg21 binding site, we generated a quadruple mutant called Atg16(4M) with the mutations S105N, T107A, E109G and N111I (figure 3*b*). As expected, introduction of this quadruple mutation into the GAD-Atg16-PTAP fusion prevents binding to GBD-Atg21 without affecting PTAP-mediated interaction with LexA-TSG101 or interaction with GBD fusions to Atg5 and Atg16 (figure 3*b*). We further tested additional mutations that can potentially disrupt Atg16 dimerization to confirm the relationship between Atg16 dimerization and Atg21 binding. Since three of the dimerization mutants (L75P, L117P and L120P) identified in the screen are proline substitutions that could disrupt the helical structure of the CCD, we analysed the effect of aspartate substitutions L117D-L120D. In addition, we tested the L85A-I89A-L99A dimerization mutant characterized in a previous work [36]. In both cases, these mutations block both Atg16 dimerization and Atg21 binding without preventing Atg5 binding and PTAP-mediated interaction with TSG101, thus confirming the screening results (figure 3*b*).

Then, we further showed that the Atg16 quadruple mutant does not bind Atg21 *in vivo* in co-immunoprecipitation assays from cell extracts (figure 3*c*). Moreover, we confirmed that lack of binding is also observed with the Atg21 quadruple mutant and one of the Atg16 dimerization mutants (N110D). Analysis of Ape1 and GFP-Atg8 processing shows that, as with Atg21, the quadruple mutation in Atg16 has a greater effect than one single mutation (N111I) and appear to block selective and bulk autophagy to the same extent as one of the dimer interface mutation (N110D) (figure 3*d*). To more accurately compare the effect of these mutations on bulk autophagy, we used the ALP assay (figure 3*e*). Consistent with the results obtained with GFP-Atg8, the N110D substitution that prevents Atg16 dimerization has a greater effect than one of the substitutions in the Atg21 binding site (N111I). The quadruple mutant (4M) has an even greater effect although residual autophagy is observed which is not detected in the *Δatg16* mutant. This residual flux, which is also detected in a *Δatg21* mutant, is likely due to alternative mechanisms of recruitment of the E3 complex to the phagophore membrane [22].

### 2.3. Analysis of the Atg21: Atg16 CCD interface

**Figure 4.**
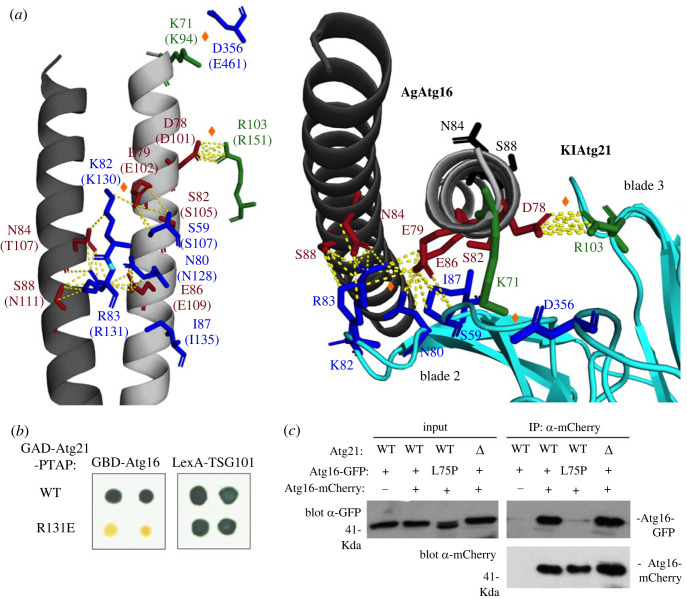
Model for Atg21 binding to the Atg16 dimer. (*a*) Illustration of the binding interface in the AgAtg16-KlAtg21 complex (PDB:6RGO) by using PyMOL. Left: Structure of *A gossypii* Atg16 coiled-coil domain (grey) showing the mutated residues identified by reverse two-hybrid screening in AgAtg16 (highlighted in red) and in one of the KlAtg21 monomers (shown in blue). KlAtg21 blades 2, 3 and 7 are not shown and only the mutated residues in Atg21 are shown for clarity. The corresponding residues in *S. cerevisiae* are shown below in parentheses. Two additional binding residues identified in a previous work are shown in green, and orange lozenges highlight three salt bridges reported in the same work [19]. Potential contacts between residues are shown as yellow dotted lines and were determined using PyMOL as any contact less than 4.0 Å. Right: View from the N-terminus of the AgAtg16 CCD. In this picture, KlAtg21 blades 2, 3 and 7 are shown in light blue. The Asn84 and Ser88 residues in AgAtg16 potentially involved in the symmetrical binding site on the opposite side of the CCD are shown in black. (*b*) Two-hybrid analysis of the Atg21-R131E mutant. The indicated GAD-Atg21-PTAP fusions were tested for two-hybrid interaction with GBD-Atg16 or LexA-TSG101 in strains Y187 (GBD) or CTY10-5d (LexA). Positives interactions were detected by β-galactosidase lift filter assays. (*c*) Analysis of Atg16 dimerization by coimmunoprecipitation. OVY552 (*atg16Δ*) and OVY551 (*atg16Δ atg21Δ*) cotransformed with pmAtg16-mCherry (+) or vector control pRS425 (–) and pmAtg16-GFP (+) or the indicated mutant derivative was grown to mid-log phase and treated with rapamycin for 120 min. Anti-mCherry-immunoprecipitated protein extracts were immunoblotted with anti-GFP or anti-mCherry Ab. Input represents 5% of protein extracts.

In the course of this study, the crystal structure of Atg21 from *K. lactis* (*Kl*Atg21) in complex with the Atg16 CCD from *A. gossypii* (*Ag*Atg16) was reported [19]. We used these structural data to find out whether the residues identified by reverse two-hybrid selection are involved in the binding interface between these two proteins. In the crystal structure, the dimeric Atg16 CCD binds two Atg21 molecules, one on each side of the CCD [19]. It should be noted that in this previous work, because of limited resolution of experimental data, one of the Atg21 molecules was subjected to Rosetta energy- and density-based optimization to improve the structural resolution of the complex [19]. However, our results best fit the interface involving the Atg21 molecule that was not further refined. Remarkably, with the exception of the amino acids involved in Atg16 dimerization, all of the residues identified by reverse two-hybrid selection in Atg16 and Atg21 are located at the binding interface between the two proteins and most of them are predicted to make intermolecular contacts. Moreover, and in agreement with the fact that Atg16 dimerization appears necessary for Atg21 binding, these potential contacts involve residues in both helices of the Atg16 CCD (figure 4*a*). In particular, two of these residues (Asn84 and Ser88 in AgAtg16) lie on the opposite face of the helix from the other residues and can only make contact with the same molecule of Atg21 if they are located on the other helix of Atg16 (figure 4*a*, right side). This provides two symmetrical Atg21-binding sites on either side of the CCD, which is consistent with the crystal structure of the complex formed by the Atg16 CCD and two Atg21 molecules [19]. Interestingly, one of the Atg21 residues identified by reverse two-hybrid selection (Lys82 in KlAtg21) can make potential contacts with residues in both helices of Atg16 (Asn84 and Glu79 in AgAtg16) and we observed that the same is true for the neighbouring residue (Arg83 in KlAtg21 and Asn84, Glu86 and Ser88 in AgAtg16) (figure 4*a*), although we did not identify mutations at this residue (Arg131 in ScAtg21) in the reverse two-hybrid screen. However, we found that the R131E substitution, like the K130E substitution identified in the screen, prevents Atg21-Atg16 two-hybrid interaction (figure 4*b*), confirming the importance of this additional residue in Atg21 binding to Atg16.

Our model in which both Atg16 helices contribute to binding to one Atg21 molecule is similar to the structure of the ATG16L1-RAB33B complex [45]. It has been suggested that RAB33B binding might stabilize the dimeric structure of ATG16L1. However, coimmunoprecipitation assays of cell extract show that the absence of Atg21 does not affect the stability of the dimeric complex formed by Atg16-GFP and Atg16-mCherry (figure 4*c*). By contrast, and as expected, coimmunoprecipitation is abolished by the L75P substitution that prevents Atg16 dimerization in two-hybrid assays (figure 4*c*).

### 2.4. The same residues in Atg21 and human WIPI2b mediate Atg16/ATG16L1 binding

**Figure 5.**
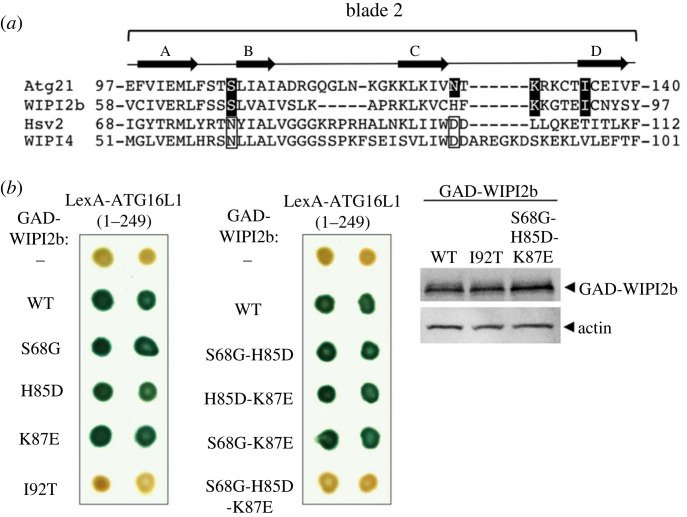
Identification of mutations in WIPI2b that prevent ATG16L1 binding. (*a*) T-Coffee alignment of the propeller blade 2 in yeast PROPPINs Atg21 and Hsv2 and the corresponding human homologues WIPI2b and WIPI4. The *β*-stranded antiparallel *β*-sheets (A–D) are shown at the top. Mutated residues in Atg21 that prevent Atg16 binding are shaded in black, and three of these residues are conserved in WIPI2b. The two conserved residues in Hsv2 and WIPI4 required for Atg2 binding and mutated in BPAN patients are indicated by open boxes. (*b*) Two-hybrid interaction between WIPI2b and the N-terminal region (aa 1–249) of ATG16L1. Left: GAD-WIPI2b (WT) and the indicated mutant derivatives were tested for two-hybrid interaction with LexA-ATG16L1(1–249) in strain CTY10-5d. Positive interactions were detected by β-galactosidase lift filter assays. Right: Protein extracts from the indicated two-hybrid transformants were immunoblotted with anti-actin and anti-HA Ab to detect HA-tagged GAD-WIPI2b fusions.

The mechanism of recruitment of the E3 complex to the phagophore membrane is evolutionarily conserved and is mediated in mammals by ATG16L1 binding to the Atg21 homologue WIPI2b/d. Remarkably, three of the four residues identified by reverse two-hybrid selection in Atg21 blade 2 are conserved in WIPI2b (figure 5*a*), raising the possibility that these residues also mediate ATG16L1 binding. To determine whether Atg21 and WIPI2b use the same set of residues to bind Atg16 and ATG16L1, we introduced into WIPI2b the mutations identified in Atg21 and analysed their effect on the two-hybrid interaction with ATG16L1. We used a truncated derivative of ATG16L1 (1–249) containing the WIPI2b-binding region [31]. We observed that one of the substitutions in WIPI2b (I92T) prevents binding to ATG16L1(1–249), whereas the combination of the other three (S68G-H85D-K87E) is necessary to obtain the same inhibitory effect (figure 5*b*). These results demonstrate that the same residues in Atg21 and WIPI2b mediate the interaction with Atg16 and ATG16L1 respectively.

### 2.5. Comparative analysis of the Atg21 and WIPI2 binding sites in Atg16 and ATG16L1

**Figure 6.**
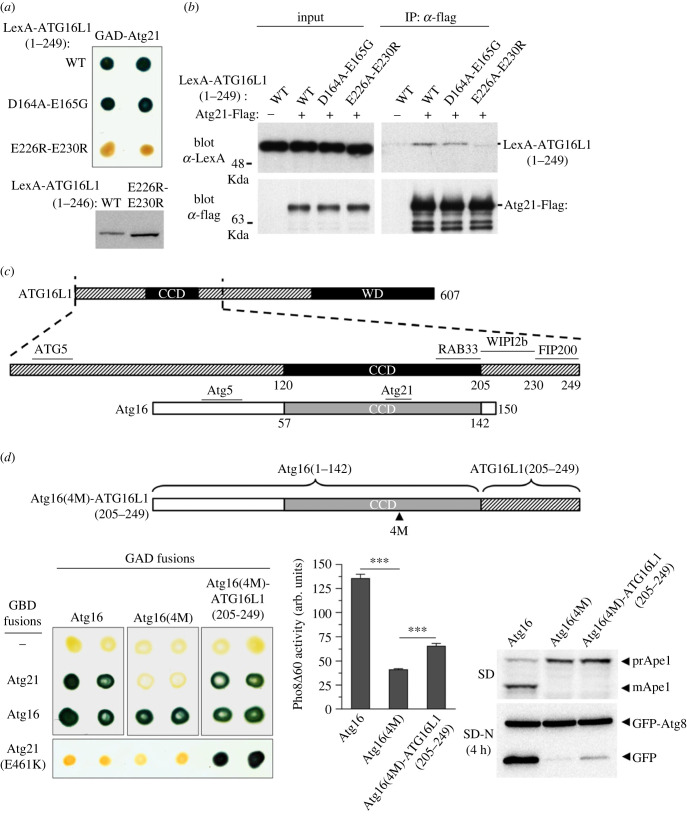
Comparative analysis of the binding mechanisms of yeast Atg21 and human WIPI2b to Atg16/ATG16L1. (*a*) Two-hybrid interaction of yeast Atg21 with human ATG16L1. (Top) GAD-Atg21 was tested for two-hybrid interaction with LexA-ATG16L1(1–249) and the indicated mutant derivatives in strain CTY10-5d. (Bottom) Protein extracts from the two-hybrid transformants were immunoblotted with anti-LexA to detect LexA-ATG16L1(1–249) fusions. (*b*) Coimmunoprecipitation of ATG16L1(1–249) and Atg21 from yeast cell extracts. OVY551 (*atg16Δ atg21Δ*) cotransformed with pmAtg21-FLAG (+) or vector control pRS425 (−) and pLexA-ATG16L1(1–249) (WT) or the indicated mutant derivatives was grown to mid-log phase. Cross-linking reagent DSP was used prior to lysis and anti-Flag-immunoprecipitated protein extracts were immunoblotted with anti-GFP (90% of the total precipitates) or anti-Flag (10% of the total precipitates) Ab. Input represents 0.5% of protein extracts. (*c*) Domain architecture of human ATG16L1 and yeast Atg16. Shown at the top are positions of the coiled-coil (CCD) and WD domains in ATG16L1. Shown bellow is an enlarged view of the ATG16L1 N-terminal region in comparison to the much shorter yeast Atg16, which lacks the C-terminal WD sequence. The binding sites for ATG5, RAB33, WIPI2b and FIP200 in human ATG16L1, and for Atg5 and Atg21 in yeast Atg16 are indicated. (*d*) Characterization of a chimeric construct of yeast Atg16 and human ATG16L1. Shown at the top is a scheme of the chimeric construct containing the N-terminal region and CCD of yeast Atg16 and the sequence containing the WIPI2b and FIP200 binding sites of human ATG16L1. In this construct, Atg16 CCD contains the quadruple mutation (4M) that prevents Atg21 binding. Left: two-hybrid assays. GBD-Atg21 and GBD-Atg16 fusions were assayed for two-hybrid interaction in strain Y187 with GAD-Atg16, the 4M mutant derivative, and the chimeric construct containing a C-terminal ATG16L1 sequence. Positive interactions were detected by β-galactosidase lift filter assays. Middle: Pho8*Δ*60 assay. OVY417 (*PHO8Δ60 atg16Δ*) was transformed with pAtg16 or the indicated mutant derivatives. Cells grown to mid-log phase were starved 4 h in SD-N medium and the Pho8*Δ*60 assay was performed. The mean values are shown with standard deviation (*n* = 3). All differences are statistically significant (one-way ANOVA). ***p > 0.001. Right: OVY383 (*atg16Δ*) was cotransformed with pGFP-Atg8 and either pAtg16 or the indicated mutant derivatives. Cells were grown to mid-log phase (SD) or starved 4 h in SD-N medium (SD-N). Protein extracts were immunoblotted with anti-Ape1 to detect the precursor (prApe1) and mature form (mApe1) of Ape1, or anti-GFP to detect GFP-Atg8 and free GFP.

The conservation of the Atg16/ATG16L1 binding residues in Atg21 and WIPI2b would suggest that the two PROPPINs probably interact with a conserved region in Atg16 and ATG16L1. However, previous studies have shown that, unlike Atg21, WIPI2b binds to a sequence downstream of the CCD in ATG16L1, which is missing in the much shorter yeast protein [31]. Interestingly, although Atg21 and WIPI2b recognize different regions in Atg16 and ATG16L1, yeast Atg21 can bind to human ATG16L1 [33]. It has been suggested that this interaction is due to the conservation in ATG16L1 of residues Asp101 and Glu102 in Atg16 (Asp164 and Glu165 in ATG16L1), which are part of the Atg21 binding site [32,33]. However, while the D101A and E102G substitutions in Atg16 preclude Atg21 binding (figure 2*a*), the introduction of the corresponding mutations in ATG16L1 (D164A-E165G) do not prevent the interaction with Atg21 in both two-hybrid and coimunoprecipitation assays (figure 6*a* and *b*). Given this result, we considered the possibility that Atg21 binds to the same motif as WIPI2b in ATG16L1, downstream of the CCD (figure 6*c*). Previous studies have identified two mutations in ATG16L1 (E226R-E230R) that prevent WIPI2b binding [31]. We found that these mutations also prevent both two-hybrid binding and coimmunopreciptation with Atg21 (figure 6*a* and *b*), demonstrating that Atg21 can recognize the WIPI2b binding site in ATG16L1, even though the equivalent region is absent in the yeast protein.

We then considered the possibility of restoring the interaction of the Atg16(4M) quadruple mutant with Atg21 by a C-terminal fusion with the WIPI2b binding sequence of ATG16L1 (figure 6*d*). Two-hybrid assays confirmed the interaction of Atg21 with the chimeric protein Atg16(4M)-ATG16L1(205–249) (figure 6*d*, left side). In contrast to wild-type Atg16, this fusion protein also binds to Atg21(E461K), demonstrating that this interaction is independent of the binding site on Atg21 located in blade 7 (figure 6*d*, left side). This result is consistent with the fact that the N-terminal to C-terminal orientation of the WIPI2b binding site in ATG16L1 is reversed with respect to the Atg21 binding site in Atg16 [32], therefore preventing contact with the E461 residue in blade 7. We next sought to assess whether this C-terminal fusion also restores the autophagic flux defect of the Atg16(4M) mutant in the ALP assay. We found that the chimeric protein significantly increases autophagic flux compared to the Atg16(4M) mutant (figure 6*d*, middle graph), thus indicating that C-terminal fusion of the WIPI2b binding sequence of ATG16L1 to Atg16(4M) not only allows Atg21 binding but also partially restores the function of this protein in autophagy. However, autophagic activity remains low in this strain, as it was not detectable in Ape1 maturation assay and barely detectable in GFP-Atg8 processing assay, which is less sensitive than the ALP assay (figure 6*d*, right side). A possible explanation is that the binding sites of Atg21 and WIPI2 on Atg16 and ATG16L1 are reversed [32] and, consequently, the positioning of the E3 complex with the chimeric protein may result in defective lipidation.

## 3. Discussion

In this study, we used the reverse double two-hybrid system (RD2H) to identify the residues in Atg21 and Atg16 that are critical for protein–protein interaction. Overall, our results, which rely largely on the yeast two-hybrid assay, are consistent with the characterization of the crystal structure of Atg21 from *K. lactis* in complex with the CCD of Atg16 from *A. gossypii*, which showed that three salt bridges as well as hydrophobic interactions stabilize this complex [19]. By using the reverse two-hybrid selection, we identified four of the six residues involved in the ionic interactions (Asp101 and Glu102 in Atg16, and Lys130 and Glu461 in Atg21) and a residue in Atg21 (Ile135) that makes potential contacts with Val112 in Atg16, one of the three residues involved in hydrophobic interactions [19].

The three salt bridges previously described involve residues in blade 2 (Lys130), blade 3 (Arg151) and blade 7 (Glu461) of Atg21 [19]. Our screen identified Lys130 and Glu461, as well as additional residues in blade 2 (Ser127, Asn128 and Ile135), the first two forming potential polar interactions with residues Glu102 and Glu109 in Atg16. However, we did not identify residues in blade 3, while we confirmed that the R151E substitution in Atg21 prevents two-hybrid binding to Atg16 (data not shown). This result indicates that our screen was not saturating, although some mutations in blade 2 were isolated repeatedly. It is likely due to the mutational bias associated with error-prone PCR methods, which has already been observed with the Taq polymerase in a previous work [48].

All the residues identified in Atg21 are adjacent residues in blade 2 except Glu461, which is further away in blade 7. We found that multiple mutations of the adjacent residues are required to fully inactivate the Atg16 binding site in blade 2 and thus impair autophagy. By contrast, mutation of residue Glu461, which forms a salt bridge with Lys94 in Atg16 [19], is sufficient to reduce the autophagic flux, suggesting that the binding site in blade 7 does not involve multiple contacts like the site in blade 2. Surprisingly, all residues identified in blade 2 of Atg21 are conserved in the PROPPIN Atg18, although this protein does not bind to Atg16 (data not shown). By contrast, the C-terminal regions of Atg21 and Atg18 are poorly conserved and residue Glu461 in Atg21 corresponds to Ile397 in Atg18, which could contribute to the lack of interaction with Atg16, as it does not allow formation of the corresponding salt bridge.

Unexpectedly, our results support the idea that Atg16 dimerization is necessary for Atg21 binding since the Atg16 residues identified by reverse two-hybrid selection are part of the Atg21 binding site or part of the dimer interface. Furthermore, we confirmed the relationship between Atg16 dimerization and Atg21 binding by showing that a dimerization mutant (L85A-I89A-L99A) characterized in a previous work [36] also prevents Atg21 binding. These results would imply that anchoring of Atg16 on membranes via the C-terminal amphipathic helix, necessary for dimerization [36], precedes Atg21 binding. Two of the dimerization mutants identified in the screen (L117P and L120P) are located in this amphipathic helix, so it is possible that a defect in membrane association of these two mutants contributes to impaired autophagic flux.

Mutated residues at the dimer interface are all proline substitutions except N110D. It is likely that the formation of the coiled-coil domain is robust to single mutations and that proline-induced local disruption in the helical structure is necessary to destabilize the dimeric CCD. Furthermore, we show that a double aspartate substitution (L117D-L120D) of two of the proline-substituted residues also prevents Atg16 dimerization and Atg21 binding. Conversely, the N110D substitution directly affects the coiled coil region containing the Atg21 binding site, although this residue does not make any contact with Atg21 in the crystal structure of the Atg21-Atg16 complex. On the other hand, we found that the Atg21 binding site in Atg16 is not limited to the previously identified residues Asp101 and Glu102 [17,35] and also includes residues Ser105, Thr107, Glu109 and Asn111, the last three making putative polar or ionic contacts with residue Arg131 of Atg21. Strikingly, these four residues are located on opposite faces of the CCD and can only make contact with the same Atg21 molecule if they belong to different helices. Notably, residues Lys130 and specifically Arg131 of Atg21 make putative contacts with residues from both helices of Atg16. These results further support the idea that Atg16 dimerization is necessary to form the Atg21 binding site. Furthermore, this provides two symmetric binding sites of Atg21 on either side of the CCD, in agreement with the crystal structure of the complex formed by the CCD of Atg16 and two molecules of Atg21 [19]. Our model is different from the model inferred from the crystal structure, in which each Atg21 molecule interacts with one of the CCD helices, which could be due to the refinement using Rosetta to improve the structural resolution of the complex [19]. Notably, binding of RAB33B to the ATG16L1 CCD also involves residues in both helices of ATG16L1 [45].

Remarkably, our results indicate that the same residues in blade 2 of Atg21 and WIPI2b mediate the interaction with Atg16 and ATG16L1. By contrast, these residues are not conserved in WIPI4 or its closest homologue in yeast Hsv2, consistent with the interaction of these proteins with ATG2A/Atg2 and not ATG16L1/Atg16 (figure 5*a*). Strikingly, mutations of two of the residues at the same position in WIPI4 and Hsv2, which have been identified in patients with the neurodegenerative disease BPAN, prevent the interaction between Hsv2/WIPI4 and Atg2/ATG2A [16]. These findings indicate that Atg21/WIPI2b and Hsv2/WIPI4 use different amino acids at the same position to interact with Atg16/ATG16L1 or Atg2/ATG2A, in agreement with the comparative analysis of the WIPI2 and WIPI3 crystal structures [32].

Interestingly, although Atg21 and WIPI2b use the same residues to bind Atg16 and ATG16L1, the Atg21/WIPI2b binding sites in Atg16 and ATG16L1 are not conserved and are located either in the CCD in Atg16 or downstream of the CCD in ATG16L1. However, we found that Atg21 can interact with the binding site in ATG16L1, although this region is absent in the yeast protein which is much shorter and truncated downstream of the CCD. Furthermore, we show that fusion of the binding site from ATG16L1 to an Atg16 mutant that does not interact with Atg21 restores Atg21 binding and partially restores the function of this protein in autophagy. Collectively, these data would suggest that the sequence corresponding to the WIPI2b binding site in ATG16L1 and located downstream of the CCD has been lost in the yeast protein as a consequence of truncation of the C-terminal region, and replaced by a site in the CCD, without changing the binding interface on WIPI2b and Atg21. However, a recent study published during the preparation of this manuscript clarifies this issue, as it shows that there are two WIPI2b binding sites in ATG16L1, the previously reported site downstream of the CCD (WBS1) and another site in the CCD (WBS2) corresponding to the Atg21 binding site in yeast Atg16 [51]. These findings suggest that WBS1 has been lost in the yeast protein, which does not contain the C-terminal extension present in ATG16L1, and that only WBS2 has been conserved. Interestingly, the absence of WBS1 could be specific to fungi, since ATG16 in *Dictyostelium discoideum*, which belongs to a group that diverged from the animal lineage before fungi, is not truncated and contains the C-terminal WD40 repeat domain missing in the yeast protein [52]. Unexpectedly, Atg21 can interact with WBS1 in ATG16L1 but not with WBS2, even though WBS1 is absent in the yeast protein and the evolutionarily conserved binding site in yeast and mammals is WBS2. Accordingly, we found that Atg21 binding to Atg16 is prevented by D101A and E102G mutations in Atg16 whereas binding of Atg21 to ATG16L1 is not affected by the equivalent mutations (D164A-E165G) in WBS2 in ATG16L1. Additionally, the E226R-E230R mutation in WBS1 in ATG16L1 fully prevents Atg21 binding, confirming the inability of Atg21 to bind WBS2. The unexpected ability of Atg21 to bind WBS1 may be due to the fact that the same residues in WIPI2b are involved in the interaction with both WBS1 and WBS2 [51], and that Atg21 and WIPI2b use these same residues to bind WBS2. On the other hand, the lack of interaction of Atg21 with WBS2, despite being evolutionarily conserved from yeast to mammals, is consistent with the fact that, except for D101 y E102, most of the residues identified in Atg16 by reverse two-hybrid selection are not conserved in ATG16L1.

Finally, our findings demonstrate the utility of the RD2H system to identify critical residues for protein–protein interaction. One of the advantages of this method is that it allows the identification of protein interaction residues without requiring prior structural information of the protein complex. The data obtained can then be used for docking-based modelling of protein–protein interfaces or to validate known crystal structure of protein complexes or structures generated with AI systems such as Alphafold multimer. When multiple residues are involved in a binding interface, structure-based mutational analysis can be challenging as it may be difficult to determine which particular residues are most important for the interaction and which substitutions are most effective in preventing binding. RD2H is a simple method to identify each of these residues and the corresponding substitutions. The sensitivity of the two-hybrid system allows the identification of single mutations that prevent binding although, as we show, they may not be sufficient *in vivo* if they are not combined with other mutations with a similar effect. We found that the combination of mutations identified by RD2H is a powerful tool to generate mutants with a complete loss of binding capacity, which is necessary to determine the physiological relevance of a given interaction. These mutants would be difficult to identify with other methods because of the large number of possible amino acid substitutions and combinations.

## 4. Material and methods

### 4.1. Yeast strains and genetic methods

The *S. cerevisiae* strains used in this study are described in electronic supplementary material, table S1. PCR-based gene deletion with the kanMX4 and natMX4 markers was performed as described previously [53,54]. Strains expressing PHO8*Δ*60 from the *GPD1* promoter were generated by a PCR-based gene modification method using pYM-N15 [55]. Standard genetic methods were followed, and yeast cultures were grown in YPAD (yeast extract-peptone-adenine-dextrose) or SD (synthetic dextrose) medium lacking appropriate supplements when plasmid selection was required [56]. Autophagy was induced by nitrogen starvation in SD-N medium for 4 h (0.17% yeast nitrogen base without amino acids and 2% glucose) or by rapamycin treatment for 120 min.

### 4.2. Plasmids

Plasmids used in this work are described in electronic supplementary material, table S2 and were constructed for the current study except pRS316-GFP-AUT7 [57] and pLexA-TSG101 [48]. Two-hybrid plasmids encoding Gal4 Binding Domain (GBD) or Gal4 Activation Domain (GAD) fusions to Atg5, Atg16, Atg21 and human WIPI2b were constructed by cloning the corresponding coding sequences in the BamH1 site of pGBKT7, pACT2 or pGAD424 (Clontech). pLexA-ATG16L1(1–249) was obtained by cloning the human ATG16L1 N-terminal region (codons 1 to 249) between the EcoR1 and Sal1 sites of pBTM116 [58]. pACT2-Atg21-PTAP and pGAD424-Atg16-PTAP were generated by cloning the Atg21 coding sequence lacking the last 5 aa or the Atg16 coding sequence in the BamH1 site of pACT2-PTAP [48] or the related plasmid pGAD424-PTAP, which contains the PTAP sequence in the Sal1 site of pGAD424. pAtg21-FLAG, pAtg16-HA and pHA-Atg12 expressing near-endogenous levels of C-terminally triple Flag-tagged Atg21 and triple HA-tagged Atg16 or Atg12 under the control of their native promoter and the *ADH1* terminator are derivatives of centromeric plasmids pRS315 and pRS313 [59], containing the Atg21, Atg16 or Atg12 coding sequence with 500 bp 5' sequence. pAtg16 is identical to pAtg16-HA but does not contain HA epitope. Multicopy plasmids pmAtg21-Flag, pmAtg16-GFP and pmAtg16-mCherry are pRS423 or pRS425 [60] based derivatives of pAtg21-Flag and pAtg16-HA in which the HA tag has been replaced by GFP or mCherry. pGAD424-Atg16(4M)-ATG16L1(205–249) and pAtg16(4M)-ATG16L1(205–249) are derivatives of pGAD424-Atg16-PTAP or pAtg16-HA containing four mutations in Atg16 (S105N-T107A-E109G-N111I) and in which the fragment containing the last 8 aa of the Atg16 coding sequence and the PTAP or HA sequences have been replaced by gap repair with a ATG16L1 fragment (codons 205 to 249). pMYC-Atg8 is a pRS316-GFP-AUT7 derivative in which the GFP sequence has been replaced by the MYC epitope. Missense mutations in Atg16, Atg21, ATG16L1 and WIPI2b were obtained by random PCR mutagenesis (reverse two-hybrid screen) or site-directed mutagenesis.

### 4.3. Yeast two-hybrid techniques

The *S. cerevisiae* strains used for two-hybrid assays were Y187 and CTY10–5d (electronic supplementary material, table S1). Two-hybrid interactions were detected by X-gal filter assays as described previously [61] and developed for 4 h. Eight independent transformants were tested of which two representative are shown.

Reverse two hybrid screens to identify mutations in Atg16 and Atg21 that disrupt Atg16-Atg21 binding were performed as described previously [48]. Briefly, random mutagenesis of the Atg21 and Atg16 sequences was carried out using pACT2-Atg21-PTAP as template and the Taq polymerase with 30 rounds of PCR (94°C for 30 s, 55°C for 30 s, and 72°C for 2 min), or pGAD424-Atg16-PTAP and the Kapa2G polymerase with 35 rounds of PCR (95°C for 15 s, 55°C for 15 s, and 72°C for 30 s). Primers used for mutagenic PCR were OV621 5′-CAC TGT CAC CTG GTT GGA CGG-3′ and OV622 5′-CTA TAG ATC AGA GGT TAC ATGGC-3′, which amplify a PCR product containing the Atg21-PTAP and Atg16-PTAP fusion flanked by 5' and 3' sequences identical to the gapped vector pACT2 digested with Nco1 and Xho1. The OVY216 strain was first transformed with the bait constructs pGBKT7-Atg16 or pGBKT7-Atg21 and the resulting transformants were then co-transformed with the gapped vector pACT2 and the mutagenic PCR product containing the Atg21-PTAP or Atg16-PTAP sequences, respectively, to allow gap-repair cloning of pACT2 based plasmids expressing randomly mutated Atg21-PTAP or Atg16-PTAP. Transformants were simultaneously selected for Ura- and His + phenotypes as described previously [48] and mutated plasmids were recovered from 13 (Atg21 screen) and 15 (Atg16 screen) large colonies. Isolated plasmids were cotransformed with pGBKT7-Atg16 (Atg21 screen) and pGBKT7-Atg21 (Atg16 screen) into strain Y187, or with pLexA-TSG101 into strain CTY10–5d, to confirm that mutations in Atg16-PTAP or Atg21-PTAP disrupt Atg16-Atg21 binding but do not truncate the protein and therefore do not impair the PTAP-mediated interaction with LexA-TSG101. All mutants behaved as expected, although we observed that the GAD-Atg16-PTAP fusion caused toxicity and slow growth of yeast transformants. Two-hybrid results were confirmed using pGAD424, a low level expression derivative of pACT2 that does not cause toxicity, to express the mutated GAD-Atg16-PTAP fusions. Finally, loss of binding mutations in Atg16 and Atg21 were identified by DNA sequencing. Two of the Atg21 mutants containing several mutations were not further characterized.

### 4.4. Immunoblot analysis

Yeast protein extracts prepared by the NaOH/TCA lysis method [62] were analysed by SDS/PAGE and immunoblotting with anti-Flag (M2, Sigma-Aldrich), anti-GFP (G-1544; Sigma-Aldrich), anti-mCherry (68088-1-Ig, Proteintech), anti-HA (3F10, Roche), anti-MYC (9E10, Santa Cruz) and anti-Ape1 (YH-16, Santa Cruz) antibodies. Immunoblots were developed with ECL reagents (Amersham).

Analysis of Atg8 lipidation was performed in a *Δatg18* background to improve detection of lipidated Atg8, which accumulates in this mutant [20].

Coimmunoprecipitation of Atg21-FLAG and Atg16-GFP or LexA-ATG16L1(1–249) was achieved by using a protocol with the DSP cross-linker previously described in [63]. In addition, coimmunoprecipitation of Atg21-FLAG and Atg16-GFP was performed in a *Δatg18* strain treated with 200 ng ml^−1^ rapamycin for 2 h to induce autophagy. In these conditions, phagophore elongation is halted, which may increase the stability of membrane bound Atg21-Atg16 complex. Briefly, cells grown to mid-log phase were harvested and treated with 2 mM DSP in lysis buffer (25 mM Hepes pH 7.5, 150 mM NaCl, 1 mM EDTA) for 2 h at 4°C and cross-linking reaction was stopped with 100 mM Tris-Hcl, pH 7.5. Following the addition of COMPLETE protease inhibitor mixture (Roche) and Triton X-100 to 1% final, cells were disrupted with glass beads 10 × 10 s. Protein extracts were centrifuged at 13 000 g for 5 min at 4°C and the supernatant was diluted to 0.33% Triton-X100 with lysis buffer and incubated with 10 µl of DYKDDDDK Fab-Trap Agarose (ChromoTek) for 1 h on a rotating wheel. Resin was washed 4 times with lysis buffer containing 0.1% Triton X-100. Immunoprecipitated extracts were analysed by 7.5% SDS/PAGE and immunoblotting with anti-Flag and anti-GFP antibodies. Antibody detection was performed as described above.

Coimmunoprecipitation of Atg16-mCherry and Atg16-GFP was performed by using a protocol described in [22]. Briefly, cells grown to mid-log phase in SD medium were harvested and first treated with 10 mM dithiothreitol in 0.1 M Tris-HCl pH 8, and then with 0.1 mg ml^−1^ zymolyase 100T (USBiological) in 0.5x YPAD containing 1 M sorbitol at 30°C for 45 min to generate spheroplasts. Spheroplasts were washed twice with 20 mM HEPES-KOH (pH7.2) containing 1.2 M sorbitol and then incubated in 0.5x YPAD containing 1 M sorbitol and 400 ng ml^−1^ rapamycin at 30°C for 30 min to activate autophagy. Spheroplasts were pelleted and solubilized in IP buffer (50 mM HEPES pH 7.5, 150 mM NaCl, 10% Glycerol, 2.5 mM NaF and COMPLETE protease inhibitor) by Dounce homogenization. N-dodecyl-β-maltoside (DDM) was added to 1% final and lysate was incubated at 4°C for 30 min. Following centrifugation, the supernatant was incubated with 10 µl of RFP-Trap Agarose (ChromoTek) and rotated at 4°C for 1 h. Resin was washed 3 times with IP buffer containing 0.1% DDM. Immunoprecipitated extracts were analysed as described above with anti-GFP and anti-mCherry antibodies.

### 4.5. Pho8*Δ*60 assay

Pho8*Δ*60 assays to measure the autophagic flux in yeast were performed essentially as described previously [50]. Yeast extracts were prepared from 3 OD600 equivalent of cells grown in SD medium and starved 4 h in SD-N medium. Cells were disrupted with glass beads 10 × 10 s in 400 µl of ALP buffer (100 mM Tris-Hcl, pH9, 10 mM MgCl2, 10 µM ZnSO4, 1 mM PMSF) at 4°C. Protein extracts were centrifuged at 13 000 g for 5 min at 4°C. The assay reaction was done in triplicate: 50 µl supernatant was mixed with 450 µl ALP buffer prewarmed at 30°C. After adding 5 mM p-nitrophenyl phosphate, samples were incubated at 30°C for 10 min and the reaction was stopped by addition of 500 µl of 2 M glycine, pH 11. Fluorescence emission of the product α-napthol (*λ*_ex_ = 345 and *λ*_em_ = 472) was measured using a GloMax plate reader (Promega) with a UV filter. Protein concentration of the cell extract was determined by using the Pierce BCA protein assay (Thermo Scientific).

## Data Availability

The data are provided in electronic supplementary material [64].
